# Work at night, sleep loss, fatigue, and cancer: exposure, mechanisms, and epidemiological evidence

**DOI:** 10.1016/j.jncc.2026.06.008

**Published:** 2026-06-27

**Authors:** Pierluigi Cocco

**Affiliations:** Centre for Occupational and Environmental Health, Division of Population Health, Health Services Research & Primary Care, University of Manchester, Manchester, United Kingdom

The meeting of the International Agency for Research on Cancer (IARC) Working Group (WG) for Monograph No. 124 on Night Shift Work took place in Lyon from 4 to 11 June 2019. Although a clear summary statement was not provided for experimental animal studies, the evidence for the carcinogenicity of alterations in the light–dark schedule was judged to be sufficient across different experimental animal strains and in both sexes. Mechanistic studies also yielded positive findings, demonstrating effects consistent with immunosuppression, chronic inflammation, and cell proliferation, three key carcinogenic pathways.^1^ In contrast, the evidence from human studies was considered limited, largely because of sub-optimal exposure assessment, particularly in large cohort studies, and the inconsistent results observed between cohort and case-control studies. Nonetheless, the body of literature reviewed was deemed adequate to support a final evaluation for three major cancer sites:

1. *Breast cancer*: the WG consensus was that, overall, there was a positive association between night shift work and cancer of the breast. However, because of the substantial variability in findings across studies, the possibility that bias accounted for the observed association could not be excluded with reasonable confidence.
2. *Prostate cancer*: The WG found the evidence to be suggestive of a positive association between night shift work and prostate cancer risk. Yet, given the relatively small number of studies and the lack of consistent results when comparable exposure metrics were used, chance and bias could not be ruled out with reasonable confidence.
3. *Colorectal cancer*: the WG concluded that there was suggestive evidence for an association between night shift work and cancer of the colon and rectum. Nonetheless, the limited number of studies and inconsistency in their findings meant that chance and bias could not be ruled out with reasonable confidence.

For all the other cancer sites, the WG determined that no conclusive evaluation could be made, owing to the small number of available studies, inconsistencies in reported results, or the use of inadequate methods for assessing exposure to night shift work.

The aim of the *Journal of the National Cancer Center* (JNCC) special issue on “Work at Night, Sleep Loss, Fatigue, and Cancer: Exposure, Mechanisms, and Epidemiological Evidence” was to update the current status of knowledge on the relationship between night shift work and cancer risk, and to examine in detail the potential carcinogenic mechanisms involved. The contributions to the special issue updated the epidemiological evidence for specific cancer sites and shed new mechanistic insights from experimental and human studies. Together, these papers provide a more refined understanding of how night work may influence cancer development, while also highlighting persistent methodological challenges and gaps that require further investigation.

With regard to new epidemiological evidence, a review of breast cancer studies published from 2019 onwards identified six cohort studies and four case-control studies.^2^ Overall, these new findings provided some support to the IARC’s 2019 evaluation of night shift work as a probable cause of breast cancer. However, they offer no substantial advances on key unresolved issues, including potential interaction with menopausal status, chronotype, hormonal subtypes of breast cancer, or gene-environment interactions, all of which remained inconclusive in the IARC evaluation.^1^ Accordingly, while the call for precautionary measures to protect workers’ health under conditions of uncertainty remains justified, the IARC Monograph # 124 Working Group’s recommendation for long-term follow-up of prospective cohorts or nested case-control studies continues to be valid. Such studies should be supported by precise exposure assessment and by rigorous examinations of relevant interactions and potential confounding factors.

A review of 75 high-quality studies published between 2003 and 2023 on circadian disruption and cancer of the breast, prostate, colorectum, as well as emerging evidence for additional cancer sites, examined the underlying mechanistic pathways and associated economic implications.^3^ Although the reviewed literature partially overlapped with that considered in IARC Monograph # 124, the association with cancer of the breast, prostate, and colon-rectum appeared strengthened. Moreover, new evidence has emerged for other cancer sites potentially linked to night shift work, including bladder cancer in women, chronic lymphocytic leukaemia, oesophageal cancer, and lung cancer in men. Despite some inconsistencies, exposure to light at night and prolonged engagement in night shift work have been associated with suppression of melatonin secretion, dysregulation of core circadian genes, such as *CLOCK* and *BMAL1*, reduced natural killer cell activity, and chronic inflammation driven by metabolic imbalance. These mechanisms were consistently identified as major pathways contributing to cancer risk.^3^ At the societal level, the economic burden of night shift work-related cancers appears substantial, owing to high direct and indirect healthcare costs and reduced work productivity.^3^

The IARC Monograph # 124 Working Group dedicated a specific section to ovarian cancer.^2^ Although some studies reported positive associations with certain types of rotating shift or night work, limitations such as insufficient statistical power, inconsistent exposure-response trends, and the absence of chronotype assessment were considered to preclude any definitive conclusion. In this JNCC special issue, Surti et al. highlighted that a healthy lifestyle may protect against the development and progression of ovarian cancer,^4^ whereas night shift work and the resulting sleep deprivation and circadian disruption is typically associated with unhealthy eating patterns, obesity, increased alcohol consumption, and reduced physical activity. Beyond these behavioural pathways, altered expression of circadian clock genes has been documented in ovarian tumour tissue, suggesting that circadian rhythm disruption due to irregular sleep patterns or nightshift work may elevate ovarian cancer risk. In addition, reduced circulating melatonin levels may lead to hormonal imbalances that could contribute to the development of hormone-sensitive malignancies, including ovarian cancer.^4^

Although the IARC Monograph # 124 noted that “…nightshift work could influence biological processes that increase the risk associated with exposure to xenobiotics as a result of both the circadian fluctuation in biological susceptibility to them, and the desynchronisation of the mechanisms of detoxification…”, it did not examine in detail the effects of circadian disruption on the metabolism of human carcinogens.^1^ Such effects would be expected, given the well-known circadian variation in the efficacy of pharmacotherapy, yet they have seldom been investigated. In this special issue, Heibati et al. explored the chronometabolism of benzene, toluene and xylene in 71 Bulgarian gas station attendants, monitored over 1 week, including 12-h daytime shifts and night shifts.^5^ Overall exposure levels were relatively low, and only marginally higher during daytime shifts, when urinary excretion of benzene and its biomarker trans, trans-muconic acid (t,t-MA) was consistently higher. Notably, t,t-MA increased at the end of daytime shifts, whereas no such variation was observed during night shifts, likely reflecting fewer refuelling events, and therefore lower exposure potential during the night. In contrast, urinary excretion of toluene, ethylbenzene, and xylene did not differ between daytime and night shifts, nor between the start and end of either shift type. Heibati et al. also found that glycine, serine and threonine metabolism, and ascorbate and aldarate metabolism were mostly affected in night shift workers. The first pathway is involved in protein synthesis, one-carbon metabolism, and glutathione production, which is critical for Phase II conjugation processes and cellular antioxidant defence.^5^ These same pathways emerged as affected by night shift work in a scoping review by the same group, which identified mitochondrial dysfunction, lipid remodelling, immune‑metabolic rewiring, oxidative stress, and hormonal/circadian imbalance as key mechanisms, each recognised as a hallmark of cancer.^6^

Obstructive sleep apnoea (OSA) does not entail circadian disruption; that is, it does not involve misalignment between the internal clock and the external environmental cues (zeitgebers) that entrain it to the regular light-dark cycle. Instead, anatomical conditions that narrow or obstruct the upper airways cause recurrent episodes of apnoea or hypopnoea, resulting in intermittent hypoxia and repeated arousal from sleep. Consequently, patients experience short, fragmented and non-restorative sleep, which is the primary cause of the daytime sleepiness commonly observed in individuals with OSA. In this special issue, a potential link between OSA and prostate cancer risk was highlighted, possibly mediated by biological pathways common to both conditions, including hypoxia-inducible factor 1 (HIF-1) signalling and pro-inflammatory pathways involving interleukins.^7^ These shared features may reflect a common genetic background. Indeed, a 68-gene overlap between the two conditions was identified, substantially exceeding what would be expected by chance, and implicated in 32 pathways involved in general carcinogenesis and, specifically, prostate cancer. These pathways include apoptosis, cell cycle regulation, cell proliferation, inflammation, oxidative stress, hypoxia, and cell damage.^7^

A mechanistic dimension that has received comparatively little attention thus far is the influence of circadian rhythms on the tumour micro-environment, the complex network surrounding tumour cells, comprising infiltrating immune cells, cancer-associated fibroblasts, vascular and lymphatic vessels, and additional components, including the extracellular matrix, basal membranes, growth factors, and metabolites. In this special issue, Lin et al. examined in detail the role of circadian rhythms in shaping the tumour microenvironment and elucidated how these rhythms may modulate the clinical efficacy of immune checkpoint inhibitors (ICIs).^8^ At the tumour cell level, sleep disturbances increase the production of reactive oxygen species, thereby enhancing DNA damage, while simultaneously reducing DNA repair capacity and up-regulating several proteins involved in cell cycle control and cell proliferation. Overexpression of circadian genes appears to enhance tumour cell sensitivity to DNA-damage-induced apoptosis, suggesting that circadian integrity may influence both tumour biology and therapeutic responsiveness. Circadian disruption contributes to the dysregulation of metabolic pathways that sustain tumour-cell growth and proliferation. Core circadian genes such as *CLOCK* suppress certain enzymes characteristic of cancer stem cells, which preserve functions such as self-renewal and differentiation capacities that play key roles in tumour initiation, progression, and metastasis. In addition, sleep deprivation can reduce the absolute number of natural killer cells, monocytes, and multiple lymphocyte subpopulations, while circadian disruption alters immune cell migration and infiltration, collectively impairing antitumour immunity. Further effects are observed on cancer-associated fibroblasts, which modulate tumour immune responses and progression, as well as on the extracellular matrix, metabolites, and other soluble factors within the tumour microenvironment. Taken together, these alterations profoundly influence the release, absorption, metabolism, clearance, and excretion of immune checkpoint inhibitors, thereby opening future perspectives for more effective, personalised chronotherapeutic approaches to cancer treatment.

While advancing our understanding of how night shift work may contribute to cancer development, the articles collected in this JNCC special issue have not resolved the central question of whether the elevated cancer risk observed in human studies of night shift work can be explained by bias or confounding. Shift work itself is a highly heterogeneous exposure; the generic term encompasses a wide variety of schedules, backwards- or forward-rotating systems, sequences of 2 to 7 consecutive night shifts or permanent night work, and cycles interspersed with 1 to 3 rest days. This diversity in organisational patterns may differentially influence workers’ ability to adapt to the misalignment between the internal clock and the environmental cues that entrain it to the light/dark cycle and, ultimately, to the Earth’s rotation around its own axis. However, epidemiology requires large numbers to achieve the statistical power needed to detect significant associations. To advance understanding of the relationship between night shift work and cancer risk, future studies should aim to assemble large cohorts engaged in distinct types of shift schedules with well-documented information on potential confounders and personal characteristics, such as the chronotype, collected at recruitment and throughout follow-up.

Abolishing night shift work is neither feasible nor desirable for the global economy; it is not comparable to abolishing slavery. Many workers actively prefer night work, suggesting that promoting adaptation may represent the most effective preventive strategy against circadian disruption, sleep loss, and, consequently, cancer risk among night shift workers.
